# Recent Progress in Photocatalytic Applications of Electrospun Nanofibers: A Review

**DOI:** 10.3390/molecules29204824

**Published:** 2024-10-11

**Authors:** Aigerim Serik, Nurlan Idrissov, Aibol Baratov, Alexey Dikov, Sergey Kislitsin, Chingis Daulbayev, Zhengisbek Kuspanov

**Affiliations:** 1 Department of Materials Science, Nanotechnology and Engineering Physics, Satbayev University, Almaty 050032, Kazakhstan; aigerimserik3508@gmail.com (A.S.);; 2 Institute of Nuclear Physics, Almaty 050032, Kazakhstan; dikov@inp.kz (A.D.); skislitsin@inp.kz (S.K.);; 3 Bes Saiman Group, Almaty 050057, Kazakhstan

**Keywords:** electrospinning, photocatalysis, nanofibers, hydrogen, semiconductors, wastewater treatment, CO_2_ utilization

## Abstract

Electrospun fiber-based photocatalysts demonstrate significant potential in addressing global environmental and energy challenges, primarily due to their high specific surface areas and unique properties. This review examines recent advances in the application of these materials in photocatalytic processes, with a particular focus on water splitting and hydrogen production. The principles of the electrospun method are described in detail, along with the operating parameters, material characteristics, and environmental conditions that affect the fiber formation. Additionally, the review discusses the challenges, advantages, and future prospects of photocatalysts incorporating carbon materials, metals, semiconductors, and hybrid structures with improved performance. These materials have the potential to significantly improve the efficiency of hydrogen energy production, water purification, and CO_2_ recovery, highlighting their importance in engineering sciences.

## 1. Introduction

Global environmental and energy issues have increasingly attracted attention from the scientific community due to the detrimental effects of fossil fuels on ecosystems [1]. The excessive use of oil, coal, and gas has led to a significant increase in greenhouse gas emissions, resulting in global warming and associated challenges, such as extreme weather events and rising sea levels [2,3]. For example, according to the World Health Organization (WHO), approximately 99 percent of the global population breathes air that fails to meet adequate quality standards, posing serious health risks. Each year, over 13 million people die worldwide due to air pollution and environmental causes [4].

One solution to these problems is to reduce reliance on fossil fuels and partially transition to clean renewable energy sources. A notable example of such a transition is the European Union, which, for the first time in 2021, produced 10.3% of the world’s electricity from wind and solar energy, doubling its share since 2015, when the Paris Agreement was signed [5]. In May 2022, the European Commission presented the REPowerEU plan to phase out fossil fuels, proposing an investment of EUR 210 billion in renewable energy and energy efficiency. According to this plan, the capacity of renewable energy in the EU is expected to reach 1236 GW by 2030 [6]. Similar initiatives are being adopted in other countries. For example, the United States passed legislation in 2022 aimed at reducing greenhouse gas emissions by 1 Gt, including tax credits for the production of clean hydrogen, the development of new clean technologies, air capture, and clean fuels [7].

Hydrogen, in particular, has garnered significant interest as an environmentally friendly energy carrier that is expected to play a key role in the decarbonization and electrification of major energy systems in the near future [8,9]. Its high energy density and unique properties make it suitable for a wide range of applications, including fuel cell vehicles, stationary power generation, and industrial processes. However, the existing difficulties in hydrogen production, storage, and transportation limit its widespread adoption [10,11]. Hydrogen production methods can be classified based on the hydrogen or energy source. These methods are divided into renewable [12] (e.g., wind energy [13], solar energy [14], geothermal energy [15,16]) and non-renewable [17] (e.g., fossil resources [13], coal gasification, catalytic reforming, partial oxidation) [18]. The use of solar energy to produce hydrogen has several advantages over traditional methods, including lower costs and scalability [19,20].

Photocatalytic water decomposition, discovered in 1972 [21], is a promising, efficient, and simple method for producing renewable hydrogen. However, despite numerous studies, the large-scale practical application of photocatalytic water decomposition to produce pure hydrogen remains challenging [22]. Nevertheless, ongoing research allows us to confidently discuss the prospects and competitiveness of this method for effective hydrogen production [23]. The primary issue limiting the practical application of current photocatalysts is the low quantum efficiency of solar energy conversion. This challenge can be addressed by creating hybrid composite structures based on photocatalysts, achieved through doping with various elements or combining them with the plasmonic effect of noble metals (Au, Ag, or Pt) or narrow-bandwidth semiconductors [24]. From this perspective, electrospinning is a versatile, simple, and cost-effective technique for producing composite structures with a high specific surface area-to-volume ratio and the ability to regulate their composition and morphology. Given that this method is widely used in various fields, such as water purification [25], tissue engineering [26,27], and the production and delivery of medicinal products [28,29], and its scalability, it has significant potential for developing effective photocatalytic systems. Unlike conventional photocatalysts, electrospun nanofiber photocatalysts have several advantages, such as significant specific surface area, high porosity, and the possibility of surface modification to enhance photoactivity [30].

**Figure 1:**
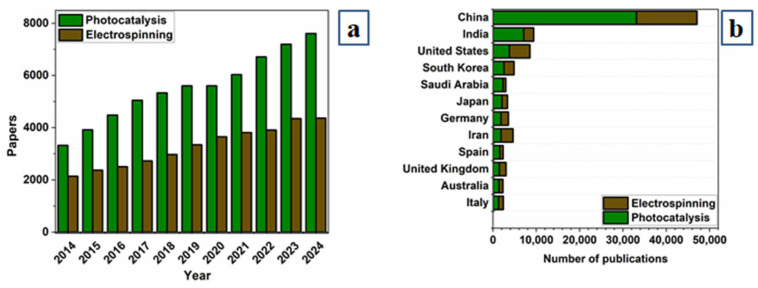
(**a**) Statistics from the Scopus database showing publication trends and keyword matches with the search string TITLE (“photocatalysis”) AND TITLE-ABS-KEY (“electrospinning”) from 2014 to 4 September 2024. (**b**) Distribution of publications by country.

Over the past decade, there has been a notable increase in research articles featuring the keywords “photocatalysis” and “electrospinning”. An analysis of publications indexed in the Scopus database from 2014 to 2024 reveals that 60,851 publications include the keyword “electrospinning” and 36,138 publications feature “photocatalysis”. Figure 1 suggest that research in this area will continue to increase. A quantitative analysis of the countries, institutions, and journals most engaged in research on photocatalysis and electrospinning shows that China is the most active, followed by India, the United States, South Korea, Japan, Germany, and Iran. In terms of citation impact, the United States leads, followed by Germany and Japan, indicating the wide use of the electrospinning method for developing composite photocatalytic systems. This review discusses the latest achievements in the formation of hybrid photocatalytic systems using electrospinning, specifically for hydrogen production. A detailed analysis of the effect of the method on the mechanism and efficiency of photocatalytic water decomposition is performed, along with the exploration of future prospects and challenges. Electrospinning is demonstrated as an efficient, low-cost approach to developing hybrid composite photocatalytic systems.

## 2. Principle of Electrospinning Method

Electrospinning is a method used to fabricate one-dimensional (1D) nanofibers from organic, inorganic, and hybrid materials [31,32]. The concept of electrospinning originated in 1600 with William Gilbert, who observed the formation of a drop of water in an electric field. In 1902, John Cooley and William Morton filed the first patents describing a prototype electrospinning rig. In 1934 and 1944, Anton Formhals filed several more patents, improving equipment to commercialize the electrospinning process [33]. The foundation of electrospinning research can be traced back to the pioneering work by Taylor from 1964 to 1969, which modelled the formation of spherical and conical shapes in polymer solutions or melt droplets under the influence of an electric field [34]. These studies initiated the development of the electrospinning method. In the early 1980s, Donaldson Co. Inc. in the United States began producing and marketing air filters fabricated using this method [33]. A new impetus for the development of electrospinning came with the introduction of electron microscopes in the early 1990s, which enabled the examination of nanoscale structures. Researchers, including Reneker and Rutledge, discovered that nanoscale fibers could be drawn from solutions of various organic polymers [35,36].

**Figure 2:**
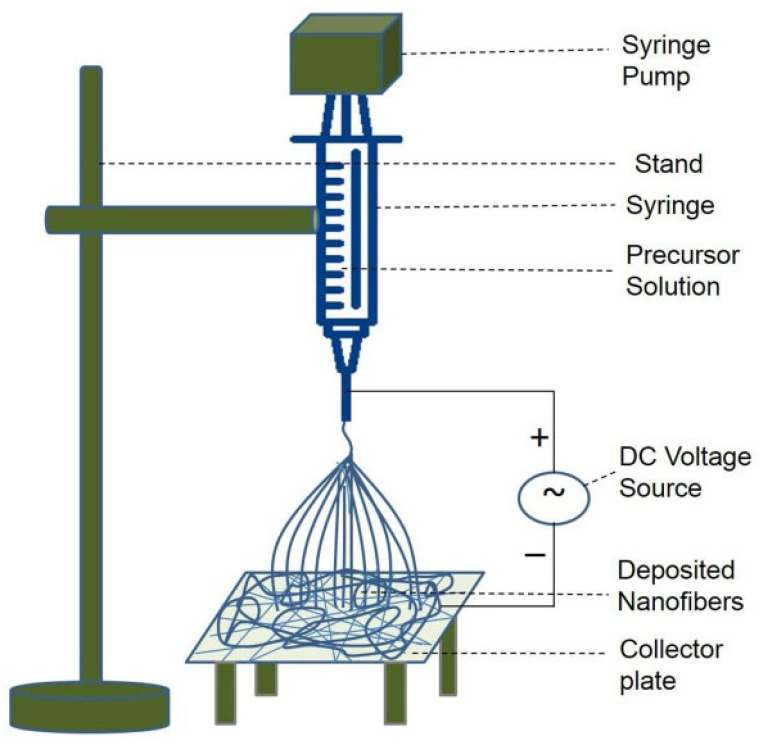
Schematic diagram of a typical electrospinning set up for preparation of nanofibers (reproduced from [37] with permission of Elsevier, 2019).

Electrospinning operates based on an electrohydrodynamic process in which a droplet of a liquid polymer is electrified to form a jet that is then stretched and elongated to produce a fiber. The typical electrospinning setup is relatively simple (Figure 2), making it accessible to most laboratories. The main elements of an electrospinning apparatus include a high-voltage power source, a needle through which the polymer solution is dispensed, and a conductive collector to collect the incoming polymer. These elements are combined into a single electrical circuit. During electrospinning, the liquid is squeezed out of the needle, forming pendant droplets due to surface tension. As the electrical voltage at the tip of the needle increases, the surface tension of the polymer solution is overcome, resulting in the formation of a Taylor cone from which a charged jet is ejected. Once the voltage is sufficient, the polymer jet rushes from the top of the cone towards the collector. The diameter of the resulting fibers depends on several factors. In air, part of the solvent evaporates, and the jet splits, depositing pure polymer on the collector as randomly or directionally aligned nanofibers with sizes ranging from nanometers or micrometers. The resulting material resembles a fibrous, porous soft fabric or a thin elastic coating. The fiber formation process can be divided into four stages [33].

Formation of a Taylor cone from which liquid is released.

Expansion of the charged jet along a straight line.

- Stretching of the jet due to the increased electric field voltage, leading to electrical bending instability.
- Solidification of the jet in the form of solid fiber(s) on a grounded collector.

The process of fiber formation by electrospinning is influenced by various operating, material, and environmental parameters.

First, the formation and deposition of fibers are affected by the electric field strength. Thinner fibers are usually formed at higher voltages, while thicker fibers or no fibers are formed at low voltages [38,39,40]. For example, increasing the voltage to 60 kV produced the thinnest nanofibers with a diameter of 190.21 ± 36.65 nm [41].

Second, the diameter and morphology of the resulting fibers are affected by the flow rate at which the polymer solution is fed into or ejected from the spinneret. To maintain a stable Taylor cone, the liquid flow rate must be adjusted continuously to match the voltage. A uniform Taylor cone results in the production of uniform nanofibers with narrow dispersion [42,43,44,45].

Third, the distance between the needle tip and the collector impacts the diameter and shape of the fibers. The minimum distance required ensures that the solvent has sufficient time to evaporate before the fiber reaches the collector. Increasing this distance results in thinner fibers. However, if the distance is too large or small, bead formation may occur [46,47].

Fourth, material properties, including the molecular weight of the polymer, viscosity of the solution, and concentration, play a crucial role in determining fiber size and structure. A higher polymer molecular weight or concentration increases fiber size. A study on the effects of PVA concentration and molecular weight (low, medium, and high) on the morphology of electrospun fibers showed that the formation of beads decreased with higher polymer concentration and molecular weight. However, at high molecular weights and concentrations, uneven and thick fibers were observed due to the increased solution viscosity [48]. High-viscosity colloidal polymer solutions can lead to an unstable jet state, resulting in fibers with a banded structure [49]. If the viscosity is too high, the solution may dry at the tip of the needle before electrospinning begins, complicating the process [47]. Notably, the choice of polymer significantly affects the formation of fibers; higher-viscosity polymer solutions typically produce fibers with larger diameters [50]. For example, using 14 wt% and 16 wt% polyvinylidene fluoride (PVDF) solutions, the 16 wt% solution yielded fewer beads and formed a membrane-like structure [51]. This is attributed to the formation of long-chain linkages in the polymer, which ensure the continuity of the jet [52].

Finally, environmental parameters such as temperature [53] and humidity significantly affect the electrospinning process and fiber morphology. Increasing the temperature to 45 °C decreased fiber diameter from 600 nm to 213 nm due to decreased solution viscosity and surface tension [54]. Similarly, increasing the relative humidity from 5.1% to 48.7% decreased fiber diameters from 253 nm to 144 nm. At humidity levels above 50%, beads formed on the fibers due to capillary instability [55].

## 3. Application of Electrospun One-Dimensional Photocatalysts

Electrospinning is a versatile, simple, and cost-effective method for producing high-quality 1D nanomaterials. However, there are certain problems associated with photocatalysts made from electrospun fibers. These problems include the recombination of charge carriers: the rapid recombination of electrons and holes reduces the efficiency of photocatalysis. Additionally, degradation of materials under ultraviolet light can lead to a loss of activity, and low quantum efficiency limits their application in visible light conditions [56].

**Figure 3:**
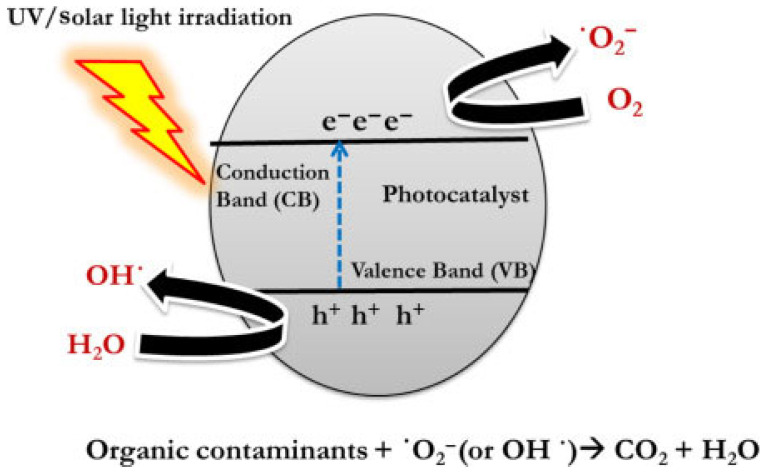
General mechanism of a photocatalytic reaction (reproduced from [58] with permission of Elsevier, 2020).

To address these issues, the surfaces of the materials can be modified. Since the photocatalytic properties of fibers primarily depend on the catalytic components, this opens up opportunities for creating 1D fibrous materials with tunable chemical composition, morphology, high specific surface area, high porosity, and varying fiber diameters. Such 1D photocatalysts are particularly promising for various applications, as they provide better light harvesting and enhanced reaction efficiency. Additionally, doping and composite formation are possible: incorporating metallic or non-metallic dopants, as well as creating composites with other semiconductors, can reduce electron–hole recombination and extend the activity of photocatalysts (Figure 3) in the visible spectrum [57].

This section discusses the unique properties of 1D nanofibers and their applications in photocatalytic water decomposition, with a focus on various photocatalyst options incorporating carbon materials, metals, and semiconductors.

### 3.1. Development of Various Electrospun Composite Nanofibers for Photocatalytic Applications

One of the most effective ways to improve the properties of 1D photocatalysts involves the incorporation of carbon materials, such as graphene, carbon nanotubes, and carbon nanoparticles. Graphene, a 2D carbon material with a honeycomb lattice structure, exhibits high electrical conductivity, sufficient mechanical strength, and good chemical stability, making it an ideal co-catalyst. It can be easily incorporated into conductive fibers by dispersing it in a polymer solution before electrospinning. The resulting fibers exhibit high electrical conductivity and a large surface area, facilitating efficient light absorption and charge separation on the catalyst surface [59,60,61]. In addition, graphene can be modified with various surfactants to enhance its adsorption efficiency. For example, reduced graphene oxide (rGO) and titanium dioxide (TiO_2_) composite fibers have shown significant improvements in the photocatalytic degradation of methyl orange compared to pure TiO_2_ [62]. Similar studies demonstrated that C/TiO_2_ nanofibers carbonized at 400 °C exhibit superior photocatalytic activity for MB degradation. This increased efficiency is attributed to the transfer of photogenerated electrons from the conduction band of TiO_2_ to the carbon during photocatalysis, leading to a more efficient separation of electrons and holes [63].

However, the primary limitation of graphene and graphene oxide as photocatalysts is their poor absorption of visible light. To address this, dye molecules that absorb visible light have been used as effective photosensitizers [64]. Positively charged dye molecules that absorb visible light are easily attracted to the negatively charged graphene oxide system due to the electrostatic forces of attraction. Studies [65] demonstrated that a photocatalyst comprising graphene oxide and positively charged dye molecules, without the inclusion of noble metals, exhibited activity two orders of magnitude higher than that of conventional TiO_2_-based catalysts.

There is also growing interest in carbon nitride, with approximately 10–12% of research on photocatalytic hydrogen production focusing on photocatalysts based on graphite-like carbon nitride (gC_3_N_4_) [66]. It is known for its stability, good light absorption (up to 460 nm) [67], large surface area and cost-effectiveness [68]. However, its photocatalytic efficiency is limited due to the high rate of electron–hole recombination, which prevents efficient reduction of H_2_ [69].

Semiconductor-based photocatalysts, particularly in the form of nanofibers, such as TiO_2_ [70], ZnO [71,72], and SrTiO_3_ [73,74,75], are known for their light-absorbing properties, high surface-area-to-volume ratio, and mechanical strength. TiO_2_ [76,77] exhibits high photocatalytic activity owing to its three main crystal structures with bandgap energies of 3.2, 3.0 and 3.1 eV. ZnO [78] has a higher light absorption capacity than TiO_2_; however, the issue of photocorrosion under UV radiation remains [79].

While these materials are extensively studied, semiconductors face challenges related to high bandgap energy and chemical stability. To improve their efficiency, many researchers have used metal or non-metal doping or combined them with other semiconductors that operate effectively under visible-light irradiation [80,81]. However, this approach limits the ability of semiconductors to absorb visible light, which constitutes the majority of the solar spectrum.

In this context, sulfide (CdS, MoS_2_) [82] and nitride (GaN, InN) [83] semiconductors have attracted considerable interest due to their small bandgaps, which enable efficient visible-light absorption. Sulfide semiconductors possess bandgaps ranging from 2.0 to 2.4 eV, enhancing their performance in the visible spectrum. However, a significant challenge with these materials is the high recombination rate of electron–hole pairs [84], which reduces their photocatalytic efficiency. Various approaches have been used to address this problem, including the addition of cocatalysts and the creation of heterojunctions [85], which aim to increase the lifetime of the charge carriers and improve photocatalytic efficiency.

Noble metals such as platinum, gold and palladium are widely used as cocatalysts for semiconductors [86,87,88,89]. These metals facilitate efficient electron transfer and reduce the probability of recombination. Additionally, they act as “traps” for electrons, extending their lifetime and increasing the number of photogenerated charge carriers.

Base metals such as iron, copper, nickel, and cobalt [90,91,92], are also used as cocatalysts. Although they are less efficient than noble metals, they offer a more cost-effective solution by creating additional active sites on the photocatalyst surface and enhancing charge-carrier dissociation.

### 3.2. Application of Electrospun Nanofibers for Hydrogen Production

Nanofibers obtained by electrospinning have recently been considered as promising candidates for applications in the photocatalytic production of solar hydrogen [73,93]. The process of photocatalytic water splitting to produce hydrogen involves three stages: light absorption leading to the formation of electron–hole pairs, charge separation and transport, followed by oxidation–reduction reactions. To efficiently split water, the photocatalyst must have a sufficient bandgap and suitable potentials for hydrogen and oxygen generation [94,95,96,97]. Therefore, there is a demand for the development of new, more efficient photocatalysts to harness solar energy effectively. A promising direction is the use of nanofiber photocatalysts, which have unique properties such as high surface area, improved charge transfer, and the ability to finely tune the morphology and structure of the material.

Electrospinning is an effective approach for synthesizing nanofiber photocatalysts [98]. The composites obtained using this method demonstrated improved characteristics compared to traditional photocatalysts, making them promising candidates for practical applications in hydrogen energy. In particular, sulfur-doped g-C_3_N_4_ nanofibers demonstrate 2.84 times higher activity in hydrogen evolution (632 μmol/h g) compared to bulk sulfur-doped g-C_3_N_4_, under similar conditions [93].

In addition, powder photocatalysts tend to settle, complicating recovery and reuse. In contrast, composite nanofiber membranes do not require mechanical stirring or ultrasound, which improves reaction stability. In particular, the ZIS/PAN membrane showed 3.7 times higher hydrogen production than ZIS powder while also simplifying the recovery process and demonstrating good stability, making it a promising solution for practical photocatalyst applications [99,100].

Carbon nanofibers obtained by electrospinning are highly effective at accumulating and transporting charge [74,101]. However, their photocatalytic efficiency may be hindered by issues such as limited active sites, rapid charge recombination, and high overpotential during hydrogen generation [93]. Possible solutions include the decoration of co-catalysts, which can increase the absorption of visible light, create more active sites, and influence charge carrier dynamics. For example, hydrogen evolution on electrospun porous TiO_2_ nanofibers with NiS and Pt cocatalysts, deposited via wet-chemical and self-assembly methods, increased 292 times compared to pure TiO_2_ nanofibers [102]. This significant increase in visible-light photocatalytic activity of the TiO_2_/NiS/Pt nanofibers upon deposition with cocatalysts can be attributed to enhanced absorption of visible light and more efficient separation of photogenerated electrons and holes.

In cocatalyst-electrospun nanofiber systems, selecting the optimal amount of cocatalyst is crucial for maximizing hydrogen generation efficiency. For example, one study [103] showed that as the content of the cocatalyst Cd_0.5_Co_0.5_S increased from 1.0 to 9.0 wt. %, the rate of hydrogen production using solar energy initially increased and then decreased. The highest efficiency of 4.55 mmol g^−1^ h^−1^ was achieved at 5.0 wt. % Cd_0.5_Co_0.5_S. The decrease in efficiency at higher concentrations was attributed to excessive cocatalyst content, which hinders charge separation, blocks active centers, and promotes the recombination of charge carriers, thus inhibiting photocatalytic water splitting [104]. As demonstrated in Table 1, the highest efficiency of photocatalytic hydrogen evolution was achieved with the nanofibers decorated with nanoparticle cocatalysts.

Photocatalytic hydrogen production based on electrospun nanofibers has great potential. However, several challenges remain for its practical implementation. These include (1) increasing the mechanical strength of nanofibers after heat treatment and (2) using safe and non-toxic solvents during electrospinning [37]. To achieve high-efficiency hydrogen generation, the development of photocatalytic material that absorbs light over a broad wavelength range and ensures efficient separation and migration of charged particles is essential. A particularly promising research direction is the fabrication of electrospun nanofibers with controlled properties and the formation of heterostructures with integrated nanoparticles, which could significantly increase their photocatalytic activity.

### 3.3. Application of Electrospun Nanofibers for Water Treatment

Composite photocatalysts synthesized by electrospinning are promising materials for purifying water from organic pollutants [87]. As previously noted, nanofibers obtained using this method exhibit a high specific surface area and a developed porous structure, enhancing their adsorption properties and access to active photocatalytic centers. This facilitates the effective destruction of stable organic compounds, such as benzene rings and carbonyl groups [110].

The most widely used photocatalysts in nanofibers are metal oxides, such as TiO_2_ and ZnO, due to their high catalytic activity and environmental safety [111]. When exposed to ultraviolet or visible light, these materials generate electrons and holes, which react with oxygen to form active oxides and hydroxyl radicals. These highly reactive species break the stable chemical bonds in pollutant molecules, such as C=C and CN bonds, mineralizing them into carbon dioxide and water. However, the bandgaps of these materials, 3.2 eV (for TiO_2_) and 3.37 eV (for ZnO), limit their absorption to ultraviolet radiation, which constitutes only 4–5% of the solar spectrum, reducing their effectiveness in the visible range [112]. In addition, the rapid recombination of photogenerated electron–hole pairs reduces the number of charge carriers involved in redox reactions, thereby diminishing the photocatalytic efficiency [113].

To increase the photosensitivity and efficiency of TiO_2_, various modifications including doping with noble metals, transition metals, rare-earth elements, and nonmetals have been explored [30]. In particular, N,F-doping of TiO_2_-δ nanofibers, developed by researchers [114], increased the degradation rate of RhB, MB and Cr(VI) dyes by 11.8, 3.2 and 2.8 times, respectively, compared to commercial TiO_2_. Doping narrows the bandgap, enabling the photocatalyst to function under visible light. This effect is attributed to the hybridization of the 2p orbitals of nitrogen with the 2p orbitals of oxygen in the valence band of TiO_2_, enhancing the separation of electron–hole pairs and reducing recombination [115,116]. Fluorine doping also improves light absorption at long wavelengths due to the similarity in ionic radii between fluorine and oxygen. The researchers further suggested that multi-element doping could provide even more improvements in photocatalytic performance compared to single-component doping.

Another promising approach to improving the photocatalytic properties of materials involves the creation of binary and ternary composites based on various metal oxides. For example, combining TiO_2_ with ZnO or Bi_2_WO_6_ leads to the formation of heterostructures that promote more efficient charge separation and broaden the spectrum of photocatalytic activity [117,118]. Ternary composites, such as NT@BMO and NT@BWO, exhibit high photocatalytic efficiency due to the synergistic interaction between components, resulting in improved adsorption of pollutants and more effective degradation of organic molecules.

Similarly, the composite NT@BWMO, a ternary heterostructure with controlled morphology and composition, was obtained by depositing BWMO on the surface of N-TiO_2_ NF [114]. The NT@BWMO-0.25, NT@BWMO-0.5 and NT@BWMO-0.75 samples synthesized at different W/Mo molar ratios (0.25, 0.75, 0.5, 0.5, and 0.75/0.25, respectively) exhibited > 99% tetracycline removal efficiency under visible-light irradiation. Among them, the NT@BWMO-0.25 sample showed the highest tetracycline degradation rate (TC) of 0.0054 min−^1^, which is 9.0, 2.5 and 1.8 times higher than that of N-TiO_2_, NT@BMO and NT@BWO, respectively. The enhanced photocatalytic activity of NT@BWMO-0.25 is attributed to the improved adsorption, optimal crystal size, a narrower bandgap, and enhanced visible-light absorption. Photoluminescence (PL) and photoelectrochemical performance (PEC) analyses confirmed that a lower tungsten ion content improved carrier mobility and increased the carrier separation rate. Radical scavenging experiments and electron paramagnetic resonance (EPR) results showed that ･O_2_^−^ radicals and h^+^ holes played a crucial role in the photocatalytic degradation process, while the influence of hydroxyl radicals was minimal. The photocatalytic activity of NT@BWMO-0.25 slightly decreased from 99.4% to 91%, indicating that the material can be reused multiple times without significant loss of efficiency.

Based on the data presented in Table 2, it can be concluded that the use of composites with multicomponent heterostructures achieves 99–100% water purification from various organic pollutants under the influence of visible light. However, the addition of g-C_3_N_4_ to nanofibers does not result in equally high photocatalytic activity. This is attributed to the limitations of the material, such as high charge recombination rates, low conductivity, and a tendency to aggregate, which reduces surface area. However, graphite-like carbon nitride (g-C_3_N_4_), consisting of tri-s-triazine structural units, is the most stable isomer, providing nanofibers with high thermal and chemical stability, thereby increasing their durability in photocatalytic applications [119].

Electrospinning allows the synthesis of materials with unique adsorption and photocatalytic properties, ensuring the complete removal of organic pollutants [120]. Nanofiber composites also exhibit high wear resistance, making them suitable for repeated use. However, scaling this technology for industrial application remains a challenge. To integrate this technology into mass production, new approaches that increase productivity without compromising material quality must be developed. Promising areas include increasing the speed of nanofiber formation, introducing multijet electrospinning, and developing cost-effective raw materials.

**Table 2:** Recent advances in electrospun fiber photocatalysts.

Year	Photocatalyst	Light Source	Pollutant	Time	Efficiency	Ref.
2021	ZnFe_2_O_4_/Ag/AgBr	UV light	Rhodamine B	100 min	86%	[121]
2020	Bimetal-PANNM	UV-visible	Reactive blue	60 min	99.99%	[122]
2021	TiO_2_@Ag@Cu_2_O	Visible light	Methylene Blue	90 min	99%	[123]
2020	ZnIn_2_S_4_/SnO_2_	Visible light	Cr(VI)	80 min	100%	[124]
2020	Co-CdSe@ECNFs	Visible light	Methylene Blue	90 min	87%	[125]
2021	ZnO	UV light	Methylene Blue	85 min	90%	[87]
2021	Bi_2_O_3_/g-C_3_N_4_	Visible light	Tetracycline	180 min	~60%	[126]
2020	Mn_2_+/ZnO	Visible light	Rhodamine B	260 min	~80%	[127]
2021	Ag_3_PO_4_-TiO_2_CNFs	Visible light	Methylene Blue	10 min	100%	[128]
2021	Ag/BiVO_4_	Visible light	Rhodamine B	20 min	~100%	[129]
2020	ZnO-TiO_2_CNFs	Visible light	Methylene Blue	120 min	~95%	[130]
2023	g-C_3_N_4_ (TiO_2_/g-C_3_N_4_@LCNFs	UV light	Rhodamine B	90 min	83.8%	[131]
2022	Chitin-modified and graphene oxide (GO) bridged TiO_2_/carbon fibers (CGTC)	Visible light	Rhodamine B	60 min	86.8%	[132]
2022	PAN/Bi_2_MoO_6_/Ti_3_C_2_ (PAN/BT)	UV-visible	Tetracycline	180 min	90.3%	[133]
2023	CuBi_2_O_4_@WO_3_	Visible light	Tetracycline hydrochloride (TCH)	120 min	70.42%	[134]

### 3.4. Application of Electrospun Nanofibers for CO_2_ Reduction

One of the most promising applications of composite photocatalysts produced via electrospinning is the reduction of CO_2_ into high-value-added products [135]. This process not only mitigates carbon dioxide emissions but also generates clean energy sources, playing a pivotal role in sustainable development and the fight against climate change. In recent years, significant research efforts have been directed towards the development and optimization of materials for photocatalytic CO_2_ reduction [136].

A particularly promising approach for enhancing photocatalytic CO_2_ reduction efficiency is the synthesis of graphene-based nanostructured materials. Graphene, due to its superior charge carrier mobility, large surface area, structural flexibility, and chemical stability, has been widely investigated for improving the photocatalytic performance of semiconductors. Specifically, the development of homogeneous ternary nanocomposites composed of graphene, noble metals, and semiconductors—without agglomeration or over-packing of grapheme—is emerging as a reliable approach for CO_2_ photoreduction [137]. Utilizing negative electric potential in the coaxial electrospinning technique enables the production of core–shell nanofibers (NFs), where metal ions concentrate beneath the rGO layer that uniformly wraps the entire fiber. These rGO monolayers, concentrated on the surface of silver (Ag), efficiently transport and collect photogenerated electrons for CO_2_ reduction while enhancing light-capturing abilities, allowing the utilization of a broader light spectrum, from ultraviolet to visible. Under visible light, rGO/Ag/TiO_2_ NFs demonstrated 25 times higher CO_2_ photoreduction efficiency, producing 4301 μmol gNF^−1^ CH_4_ in 7 h, compared to conventional semiconductor nanofibers. Similarly, graphene’s efficacy is highlighted in a study combining graphene with PVDF (polyvinylidene fluoride) and TiO_2_, which achieved a yield of 363 μmol g^−1^ in 1 h, significantly higher than the result without graphene (28.3 μmol g^−1^ in 1 h) [138]. However, it is important to note that an excessive amount of graphene may complicate the electrospinning process and reduce photocatalytic efficiency due to aggregation, which blocks incident light and hinders photocatalysis.

In addition to graphene, doping semiconductors with single-atom metal catalysts has been recognized as an effective strategy for enhancing the efficiency of photocatalytic CO_2_ reduction [139]. Titanium dioxide (TiO_2_), known for its high photocatalytic activity, thermodynamic stability, non-toxicity, and low cost, has been widely utilized in CO_2_ reduction processes [140]. Electrospun TiO_2_ nanoparticles serve as ideal substrates for the growth of secondary nanostructures, facilitating the creation of heterojunction photocatalysts. These hybrid heterojunctions improve electron–hole separation, enhance light absorption, and promote reactant activation, resulting in superior photocatalytic performance. For instance, TiO_2_ nanofibers coated with graphitic carbon nitride (gC_3_N_4_) achieved CO and CH_4_ yields of 5.19 and 1.65 μmol/g, respectively, representing a 1.8-fold increase in CO_2_ conversion performance compared to gC_3_N_4_ alone [141]. However, TiO_2_ faces limitations such as low surface active site density, a high recombination rate of photogenerated charge carriers, and limited CO_2_ capture efficiency. To overcome these challenges, various modification strategies have been explored [142].

In parallel with TiO_2_, alternative semiconductor materials have been investigated for photocatalytic CO_2_ reduction, including metal oxides (e.g., ZnO), metal chalcogenides (e.g., ZnS, CdS), perovskite halides (e.g., CsPbBr_3_), MXenes (e.g., Ti_3_C_2_), layered double hydroxides, and metal–organic frameworks [143,144,145]. However, these materials often exhibit low practical efficiency due to rapid electron–hole recombination and limited sunlight utilization. Consequently, significant efforts have been made to develop more efficient photocatalysts by manipulating their morphology, adjusting the bandgap, and introducing metals to improve performance [146].

Recently, single-atom catalysts (SACs) have gained considerable attention in various catalytic reactions due to their unique physical and chemical properties [147]. Several SAC-based photocatalysts, including Cu/CN [148] and Pt@WS_2_ [149], have been synthesized. In particular, doping TiO_2_ with noble metals significantly enhances catalytic activity through three primary mechanisms: Fermi level alignment, efficient electron trapping, and the creation of thermal catalytic sites for adsorbed molecules and reaction intermediates [123]. For instance, co-deposition of Pt on TiO_2_ has demonstrated exceptional activity in CO_2_ conversion to CH_4_, outperforming pure TiO_2_ by a factor of 10 [150]. This enhanced performance is attributed to Pt nanoparticles acting as electron traps, facilitating charge separation on the TiO_2_ surface. On the other hand, Au/TiO_2_ nanofibers exhibit lower activity in CH_4_ production but demonstrate higher CO production, highlighting new possibilities for selective control of photocatalytic reaction products.

The key parameters and results of recent studies on the photocatalytic reduction of CO_2_ into value-added products are summarized in Table 3. Based on a comparative analysis, the Ni-MoP@NCPF photocatalyst exhibits the highest efficiency in reducing CO_2_ to CO [151] (953.33 μmol g^−1^h^−1^), while the Graphene@PVDF@TiO_2_ composite [138] shows superior efficiency in CH_4_ formation (363 μmol g^−1^ h^−1^) under visible-light irradiation. When comparing photocatalysts, it is essential to consider the use of sacrificial agents, carbon and hydrogen sources, and the type of radiation.

## 4. Conclusions

In conclusion, electrospun nanofiber photocatalysts demonstrate significant potential for practical application in hydrogen energy, water purification and CO_2_ recovery. The advantages and prospects of various photocatalyst modifications, including the addition of carbon materials, metals, and semiconductors, as well as the development of hybrid structures with improved characteristics, are discussed. Due to their unique physicochemical properties, such as large specific surface area and improved charge separation, these materials can significantly improve photocatalytic efficiency. However, for widespread implementation, several technical challenges must be addressed, including increasing the mechanical strength of nanofibers post-heat treatment and using safe, non-toxic solvents during synthesis. The prospects for further development of electrospun composite materials offer broad opportunities to enhance their photocatalytic properties. Research in this area can focus on optimizing the composition and structure of nanofibers, utilizing new co-catalysts, and modifying the surface to improve the efficiency of light absorption and solar energy conversion processes. A key challenge is addressing the issues related to electron–hole recombination, as well as developing cost-effective solutions to increase the lifespan and stability of photocatalysts. These directions could significantly expand the application of electrospun materials in environmentally friendly technologies and energy-saving processes, including photocatalytic hydrogen production and water purification.

## Figures and Tables

**Table 1:** Recent research results (2022–2024) on the use of electrospun nanofiber-based photocatalysts in H_2_ production.

Year	Photocatalyst	Light Source	Sacrificial Agent	H_2_ Evolution Rate (mmol h^−1^g^−1^) and AQY	Ref.
2023	Sg-C_3_N_4_ nanofiber	Metal halide 400 W, full spectrum	20 vol.% methanol	0.632	[105]
2023	TiO_2_/NiS/Pt nanofiber	5 W blue LED light, λmax = 420 nm	50 vol.% methanol	4.411	[102]
2024	NiGa_2_O_4_/ZnIn_2_S_4_ nanofiber	300 W Xe-lamp, AM 1.5 filter, 41.7 mW/cm^2^	10 vol.% TEOA	9.292	[93]
2024	CoGa_2_O_4_/ZnIn_2_S_4_ nanofiber	300 W Xe-lamp, AM 1.5 filter, 41.7 mW/cm^2^	10 vol.% TEOA	6.283	[93]
2022	Cd0.5Co0.5S/SN-TiO2 nanofiber	300 W Xe-lamp	2.4 g Na_2_S, and 1.26 g Na_2_SO_3_ into 100 mL deionized H_2_O	4.55 and AQY of 19.01% at 410 nm	[103]
2024	ZnIn_2_S_4_/PAN nanofiber membrane	Visible light (420 nm ≤ λ ≤ 700 nm)	10 vol.% TEOA	1.836 and AQY of 1.77% at 365 nm	[99]
2023	S-scheme BaTiO_3_/Ag_2_S nanofiber	300 W Xe-lamp	Na2S (0.35 mol/L) and Na_2_SO_3_ (0.25 mol/L)	0.597	[106]
2024	In_2_S_3_–In(OH)_3_–ZnS nanofibers	5 W blue LED light (λmax = 420 nm, 41.7 mW cm^−2^)	0.1 M Na_2_S solution	0.2236	[107]
2024	C–Ni_2_P/ZnCr_2_O_4_ nanofibers	Xe lamp intensity of 350 mW cm^−2^	0.2 g Na_2_S, and 0.2 g Na_2_SO_3_ into 100 mL deionized H_2_O	0.5759 and AQY of 15.25% at 420 nm	[108]
2023	CdS NPs-decorated ZnO nanofibers	500 W Xe lamp with 425 nm band pass filter	0.35 M Na_2_S and 0.25 M Na_2_SO_3_	0.820	[109]

**Table 3:** Recent research results (2020–2024) on the use of electrospun nanofiber-based photocatalysts for CO_2_ reduction into value-added products.

Photocatalytic Nanofibers	Light Source	Reagent	Products	Reaction Rate	Ref.
g-C_3_N_4_/black titania	300 W Xe-arc lamp	CO_2_ + H_2_O + TEOA	CO and CH_4_	5.19 and 1.65 μmol/g	[141]
(rGO)-wrapped Ag/TiO_2_	500 W Xe lamp with a 400-nm long pass filter	CO_2_ + H_2_O vapor	CH_4_	4.301 μmol g^−1^	[137]
Ni-NiS/C/ZnO	350 W simulated solar Xe arc lamp, 10,117 μW cm^−2^	CO_2_ + H_2_O + NaHCO_3_	CO and CH_4_	5.86 and 1.14 μmol g^−1^ h^−1^	[152]
NiS@Ta_2_O_5_	Xe lamp, 920 mW cm^−2^	CO_2_ + H_2_O	CO and CH_4_	43.27 and 6.56 μmol g^−1^ h^−1^	[153]
TiO_2_/MoSe_2_	300 W Xe-arc lamp, 12 mW/cm^2^	CO_2_ + H_2_O + TEOA	CH_4_ and CO	174.02 and 478.46 μmol/g	[154]
Nb_2_O_5_	18 W mercury lamp, 254 nm	CO_2_ + H_2_O vapor	CO and CH_4_	8.5 and 0.55 μmol g^−1^	[155]
Ni-MoP@NCPF	300 W Xe lamp with a UVCUT 420-nm filter	CO_2_ + acetonitrile/H_2_O + TEOA	CO	953.33 μmol g^−1^h^−1^	[151]
C doped TiO_2_	300 W Xe lamp, AM 1.5 filter	CO_2_ + H_2_O + NaHCO_3_ + H_2_SO_4_	CH_4_	55.17 μmol g^−1^ h^−1^	[156]
SrTi1-xCuxO_3_-H_2_	300 W Xe lamp, (400 nm < λ < 780 nm)	CO_2_ + H_2_O	CH_3_OH	5.38 μmol g^−1^ h^−1^	[157]
Graphene@PVDF@TiO_2_	Two 300 W visible light sources (UV < 5%)	CO_2_ + H_2_O	CH_4_	363 μmol g^−1^ h^−1^	[138]
TiO_2_/MoS_2_/g-C_3_N	300 W Xe-arc lamp, 12 mW/cm^2^	CO_2_ + H_2_O + TEOA	CH_4_	21.78 μmol g^−1^	[158]

## Data Availability

Data are contained within the article.
